# 

**Published:** 1901-10

**Authors:** 


### Society Notes.

---

The West Texas District Medical Association will meet in San Antonio, October 31, 1901.

---

### The East Texas Medico-Chirurgical Society.

The East Texas Medico-Chirurgical Society will meet in Palestine, Texas, November 28th and 29th; and as business of an important nature will come before this body at that meeting, besides the usual rich program, every physician in East Texas who is interested in the medical profession, and especially the members of this society, are hereby earnestly and cordially requested to be present.

E. E. GUINN, Secretary,
Rusk, Texas.

---

### Brazos Valley Medical Association.

CALVERT, TEXAS, September 16, 1901.

DEAR DOCTOR: The next meeting of the Brazos Valley Medical Association will be held on the 12th and 13th of November, 1901, at Bryan, Texas.

You are earnestly requested to prepare a paper on a theme of your own selection. Please send your subject to Dr. W. B. Briggs, Easterly, Texas, at once.

We hope every member will be present.

DANIEL PARKER, President.

W. B. BRIGGS, Secretary.

